# Focus of Sustainable Healthy Diets Interventions in Primary School-Aged Children: A Systematic Review

**DOI:** 10.3390/nu15112460

**Published:** 2023-05-25

**Authors:** Elena Patra, Anna Kokkinopoulou, Ioannis Pagkalos

**Affiliations:** Nutrition Information Systems Laboratory (NISLAB), Department of Nutritional Sciences and Dietetics, International Hellenic University, 57001 Thessaloniki, Greece

**Keywords:** sustainable healthy diets, sustainable nutrition, sustainable food systems, primary schools, intervention, sustainable diet indicators

## Abstract

Research and public policy interest regarding Sustainable Healthy Diets (SHDs) have increased during the last decades, as nutrition recommendations and diet practices should align with growing environmental concerns. SHDs encompass sociocultural, economic and environmental components of nutrition and health and raising awareness across all these dimensions, as well as providing relevant education, especially to young children, is important for adopting SHD practices. Primary school students (5–12 years old) are often the target population for interventions, as they are considered agents of change for educating the community. The objective of this systematic review is to map the SHD indicators addressed by such interventions in order to identify gaps and opportunities for future interventions in this population. Scopus, PubMed and Web of Science were searched for available publications, following the Preferred Reporting Items for Systematic Reviews and Meta-Analysis (PRISMA 2020) methodology. After screening for eligibility, thirteen intervention studies were included and reviewed. Results showed that indicator definitions and measuring methods were not harmonized across research efforts. Implemented SHD interventions address predominantly food waste and diet quality, while social and economic indicators are underrepresented. The standardization of SHD, focusing on measurable harmonized indicators, should be a priority for policy actors in order to enable impactful research efforts. Future interventions should incorporate clear SHD indicators to raise awareness and consider the application of composite tools or indexes to evaluate outcomes and maximize impact in the community.

## 1. Introduction

In recent years, it is becoming more and more evident that the current state of our global food system is not sustainable for our environment and our health. Poor-quality nutrition has been linked to economic, sociocultural and environmental factors such as cost of diets, low-income levels, food insecurity, food availability, lack of education [1,2,3,4], low access to sanitation including clean drinking water [5], reduction of natural resources and local agriculture [6] and urbanization [7] among others. As a result, poor nutrition and unhealthy diets have resulted in worldwide rates of overweight and obesity doubling within three decades [8] while future generations seem to be already compromised; the World Health Organization’s (WHO) latest report on childhood obesity showed that almost one third of young children in Europe are already living with overweight and obesity [9,10], while the UNICEF-WHO-World Bank Joint Child Malnutrition inter-agency group estimates that almost 150 million children suffered from stunting in 2020 [11]. Therefore, tackling all forms of malnutrition is essential to ensure health, well-being and consequently, sustainable development [12], as adopting sustainable and healthy eating habits is vital for both human health and the planet [13].

As a response to the above, there is an urgent need to develop sustainable food systems (SFSs) that can deliver food security and nutrition for all in such a way that the economic, social and environmental bases to generate food security and nutrition for future generations are not compromised [14]. SFSs and sustainable diets are profitable throughout, have broad-based benefits for society and have a positive/neutral environmental impact and environmental biodiversity for present and future generations [15,16,17].

The importance of sustainable nutrition has been a focal point among policy actors, especially within the last decade, since the Food and Agriculture Organization of the United Nations (FAO) and the WHO published their joint guiding principles on Sustainable and Healthy Diets (SHD) [14]. Around the same time, the EAT-Lancet Commission on Food, Planet, Health published their report on Healthy Diets from SFS, that focused on the need for dietary shifts and sustainable food production, including greenhouse gas emissions (GHGE), cropland use, water use, nitrogen and phosphorus application and biodiversity loss [18]. The importance of sustainability comes in line with the work of the United Nations (UN) which in 2015 proposed the 17 Sustainable Development Goals (SDG) as part of the 2030 agenda for global sustainable development [19,20]. Lastly, the European Farm to Form strategy, highlights the need for SFS with a neutral or positive environmental impact, that avoid loss of biodiversity, ensure food security, nutrition and public health, access to sufficient, safe, nutritious and sustainable food, while preserving food affordability within a fair competitive trade [21].

Despite these important steps, sustainability or sustainable development can be commonly perceived as obscure terms or catchphrases if not clearly and adequately described within a specific context [22,23,24]. Consumers’ perceptions of sustainability and SHD are unclear as there seems to be a gap between consumers’ beliefs and scientific evidence about what constitutes a sustainable diet. Common misconceptions exist such as, for example, that a plant-based dietary model is enough to be consider an SHD, while other aspects such as food waste reduction and modern sustainable agriculture must be simultaneously pursued [25]. Furthermore, the environmental and health-benefits of meat-free diets are underestimated, while local food consumption is perceived overly positive when comparing it to plant-based diets [26]. Another example is that a healthy diet is necessarily perceived as environmentally friendly [27]. To avoid misconceptions and oversimplifications, robust indicators for SHDs are critical for understanding trends, setting targets and monitoring progress across national and sub-national levels [19,20].

Therefore, to achieve the goal of transitioning to sustainable nutrition and food systems, it is also necessary to describe, design and evaluate SHD standards. There is a universal consensus on the environmental, sociocultural and economic dimensions of SHD across the scientific literature [28,29,30,31] while health, nutrition and diet that are considered core components, are often referred to as the fourth dimension [32]. Several indicators of nutritional sustainability have been developed or proposed under each dimension in recent years, most of which are based on the planetary health diet reported in the EAT-Lancet report [33]. However, there is great heterogeneity between proposed indicators and the methodology that describes them. Recent systematic reviews attempted to summarize proposed indicators across the literature [28,29,30,31,34], with some of the most common indicators being water use, food waste, GHGE [28,29,31], biodiversity [30] or energy use and land use, nutrient density and adequacy, cultural affordability [31], cost of diets and even composite ones such as affordable nutrient density [35]. However, single indicators cannot adequately account for environmental impacts or fall short in considering the economic impact or ethical aspects of sustainable nutrition.

In a recent scoping review, Harrison and colleagues proposed another category, which was described as cross cutting indicators, i.e., terms that overlap across multiple aspects of SHD including scores and indexes [31]. Scoring systems and/or indexes have been widely proposed to quantify SHD [33]; examples include the Sustainable-HEalthy-Diet (SHED) index score, which is validated based on the planetary healthy diet proposed by the EAT-Lancet report, the Mediterranean Diet score, which reflects the nutritional, environmental and sociocultural aspects of sustainable diets [36], the Planetary Health Diet Index (PHDI), which is adapted from the recommendations of the planetary healthy diet proposed by the EAT-Lancet report [37] and the World Index for Sustainability and Health (WISH) score, which combines diet quality and environmental sustainability in one scoring system based on the EAT-Lancet recommendations [38]. Similarly, the French Sustainable Diet Index (SDI) includes seven indicators across environmental, nutritional, economic and sociocultural dimensions and was based on the sustainability of dietary patterns [39]. Whether research focuses on indicators or composite tools for assessment, such as indexes, there is universal agreement on the need to harmonize and establish standardization in the description and methodological measurement of SHD indicators and relevant tools [28,29,30,31,33,34].

Even though Education for Sustainable Development (ESD) is recognized as a key enabler for SDGs by UNESCO [40] recent work has found current proposed research initiatives addressing SDG4 (“inclusive and equitable quality education and lifelong learning opportunities for all” [19] to be insufficient [41,42]. Furthermore, recent studies indicate that children currently neglect the economic and social—especially cultural—dimensions of SHD [43]. Raising awareness through community education is necessary to shift consumer perceptions and achieve the SDG targets by adopting SHD practices [6,30]. In this respect, the school setting is ideal for interventions focusing on forming children’s behavior, as it affects children’s lives during their first and critical years of life, when habits are still evolving [44]. School interventions can have a broader impact as they can result in positive effects, i.e., nutrition knowledge for parents [45]. Primary school-aged interventions, in particular, have been proven successful in shaping healthy eating behaviors, preventing unhealthy weight gain, improving health parameters [46] and increasing fruit and vegetable consumption and/or nutrition-related knowledge [47] and can be effective over time [48]. Furthermore, systematic review evidence suggests that certain digital resources characterize effective digital interventions for health behavior change in adolescents [49] and that web-based interventions may be effective in promoting some sustainable diet-related outcomes in young adults [50].

Based on the above, this review aimed to explore and map the SHD indicators used to date in primary school interventions, in order to identify gaps and opportunities for SHD indicators and/or composite indexes to be used in future research in this population group.

## 2. Materials and Methods

### 2.1. Literature Search

A literature search for SHD interventions in primary schools was conducted between March and April 2023 on the PubMed, Web of Science and Scopus scientific databases, in accordance with the Preferred Reporting Items for Systematic Reviews and Meta-Analysis (PRISMA) 2020 guidelines [51]. The PRISMA Checklist for this systematic review can be found under Supplementary Material. Relevant literature was identified by searching for the following keywords with any possible combination in publication abstracts: ‘sustainable healthy diets’ OR ‘sustainable diets’ OR ‘sustainable nutrition’ OR ‘sustainable food system(s)’ AND ‘intervention’ AND ‘schools’ OR ‘students’ OR ‘adolescents’.

### 2.2. Eligibility Criteria

Criteria for the included publications were the following: (i) studies involving human populations, (ii) studies including any intervention activities and pre- and post- intervention measurements, (iii) studies in which the target population included children between 5–12 years old (age range of students in primary/elementary school across different countries), (iv) studies published in English, (v) studies published between January 2013 and March 2023 and (vi) full-length papers. Editorials, guidelines, position perspective, discussion papers, study protocols, modeling studies, abstracts and unpublished results were excluded from the review process. After duplicate publications were removed, abstracts of full publications were assessed for eligibility in this review, in accordance with the inclusion criteria.

### 2.3. Quality Assessment of the Studies

The latest version of the Mixed Methods Appraisal Tool (MMAT) was used for quality assessment of all included studies [52]. Each study is appraised only within its methodology based on five criteria rated as ‘Yes’, ‘No’, or ‘Can’t tell’. Overall study quality can range from 20% or 1* (one criterion met) to 100% or 5***** (all five criteria met). Two authors (EP and AK) independently reviewed all studies that met the eligibility criteria. Any discrepancies were resolved over consensus discussion. All included studies scored at least 3*** or 60%. Outcomes of the MMAT quality assessment can be found in Appendix A.

## 3. Results

**Figure 1:**
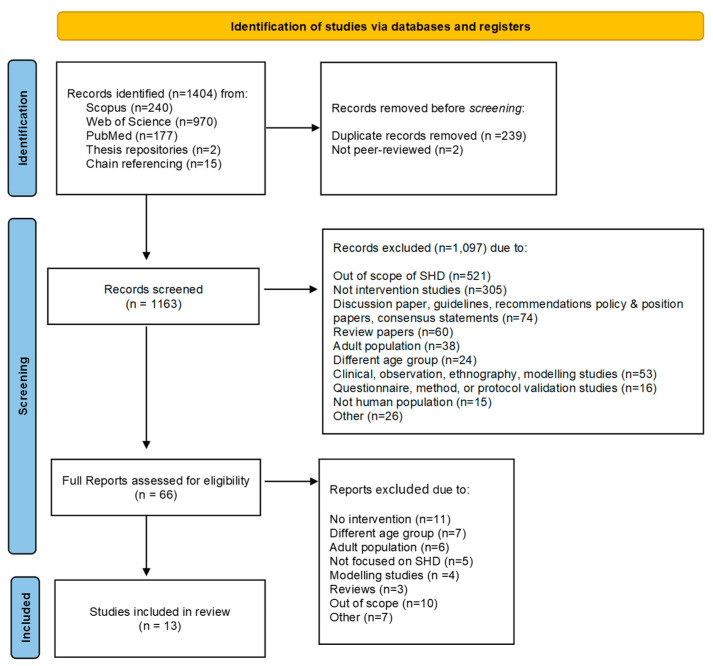
Flow diagram of the identification process, following PRISMA 2020 guidelines.

A total of 1404 records were identified through database searching, and other sources (chain referencing and thesis repositories), as can be seen in Figure 1. After removing 239 duplicate findings and 2 non-peer reviewed documents, 1163 papers were screened at title and abstract, and 1097 were excluded. Out of the 66 full text articles that were screened for eligibility, 53 were excluded. The resulting 13 articles were included in the final review.

### 3.1. Eligible Publications Main Characteristics

Publication details (title, first author, year and country) as well as basic information on the interventions (study design, sample details and age group) of the 13 reviewed articles are presented in Table 1. Most of the studies were implemented in different European countries [53,54,55,56,57,58,59], three in the USA [60,61,62], two in Asian countries [63,64] and one in Australia [65]. Studies were implemented directly in the school environment, with one exception of a community intervention in which children were accessed through their families [60].

All studies covered the ages of 3 to 18 years old with primary school-aged children being most represented across the studies. Studies with subjects under 5 years old and/or over 12 years old were included, as long as the study also included children of the targeted age target (5–12 years old). In studies where the age group or mean age was not reported, eligibility was confirmed based on school grade through national sources. Several studies did not report gender distribution in their sample demographics; when reported, male and female participation was relatively balanced in the intervention groups [56,58,60,61,63,64].

Different study designs were applied between studies. Six studies applied Randomized Controlled Trial (RCT) or Non-Randomized Controlled Trial (Non-RCT) designs [53,54,56,60,62,63], three implemented One-group Pretest Posttest Design (One group PPD) [55,59,64] and four studies applied mixed methods approaches [57,58,61,65]. Regarding participation, both the number of participating schools as well as sample size was largely different and often small or not representative of the population. Regarding sample size, we report the overall number of children who received interventions (not control groups). More specifically, four interventions implemented activities in a single school (either with one-group or control group designs) with student participation ranging between 10–109 children for those studies [58,59,62,64]. Most interventions were implemented in multiple schools (2–5 across studies) [53,55,56,57,61,62] with very large differences in sample size, ranging from *n* = 151 to *n* = 1635 in one study [55]. One study included 9 interventions schools with *n* = 235 children overall in the treatment group [63]. Finally, in one intervention, activities were implemented in the community (through families) instead of the school environment, with a participation of *n* = 106 children [60].

Finally, interventions differed considerably in terms of their duration, which varied from a short one-off session (including studies which reported minutes as a duration) to repeated exposure over a 6-month period (when taking into account all intervention activities or elapsed timed between pre- and post- intervention).

### 3.2. Intervention Cctivities

Overall, the intervention landscape was diverse in terms of implemented activities, methods and measurements of impact evaluation, as well as addressed SHD indicators, as can be seen in Table 2. Due to this diversity, we focus on the main differences or most unique approaches to highlight the scope that the interventions cover and understand gaps or opportunities for SHD indicators.

As discussed previously, intervention activities varied from one single communication session to approaches that incorporated several elements over extended periods of time. More specifically, 10 studies included some educational or pedagogic element that aimed to raise awareness on the topic of nutrition and/or sustainable agriculture aspects through nutritionist session [59], educational curriculum [58,60,61,62,63,65], teacher trainings [53,56] and classroom sessions [58,62,63]. Other approaches incorporated co-creation activities with active children participation through school gardening [60,63], poster creation based on the learnings of preceding curriculums [61], local farm visits [62] and engagement in activities with parents or guardians [53,60,65]. Some studies implemented more than one type of intervention activity and actively involved teachers and parents apart from children. In the Healthy Children, Healthy Planet (HCHP) study, researchers collaborated with the World Wide Fund for Nature (WWF) to implement a combination of teaching and parent trainings with purpose-built educational material focused on SHD for children, parents and teachers [53].

Only three interventions did not actively educate participating children on SHD issues before implementing implicit intervention activities [54,55,64]. More specifically, the Fostering Healthy and Sustainable Diets Through School Meals (OPTIMAT) study applied linear regression modeling to optimize school meal content in Swedish schools, considering three SDH indicators in their optimization model GHGE, nutritional adequacy and meal cost. They measured success by mathematically calculating changes to these indicators in the optimized menus. In addition, they considered meal satisfaction and food waste pre- and post-intervention [55]. In the Forced Vegetarian Day (FVD) study in Helsinki, researchers implemented forced choice restriction in school canteens by running days where only vegetarian meal options were available to children. The study measured the percentage of children’s participation in the FVD as well as the food waste compared to the standard school meal routine where non-vegetarian options were also available [54]. The third study was conducted in Indonesia, a country in which schools typically do not provide meals, by introducing a locally sustainable school lunch following nutritionist advice to assess the impact on children’s nutritional status, BMI and blood test results [64].

### 3.3. Impact Evaluation Methods/Measures

Different methods were applied to assess the effectiveness of respective activities (impact evaluation), even between studies that followed similar educational approaches. For example, researchers in Bhutan performed educational activities in combination with school gardening activities, measured attitudes, knowledge and change in anthropometry post intervention but did not assess changes in any SHD indicator, despite having some included as part of their intervention material on sustainable agriculture [63]. In a study in Indonesia, apart from the changes in dietary intake, blood samples were collected to measure changes in hemoglobin and hematocrit in order to assess the impact evaluation of introducing sustainable lunches in schools [64]. Eight studies applied a similar approach of measuring changes in SHD indicators, namely food waste [53,54,55,56,57,58,61,65] or specific dietary quality, such as fruit and vegetable intake [54,61] to evaluate intervention impact. In the OPTIMAT study, researchers measured success by mathematically calculating changes to these indicators in the optimized menus. In addition, they considered meal satisfaction and food waste pre- and post-intervention [55]. In the Forced Vegetarian Day (FVD) study in Helsinki, researchers implemented forced choice restriction in school canteens by running days where only vegetarian meal options were available to children. The study measured the percentage of children’s participation in the FVD as well as the food waste compared to the standard school meal routine where non-vegetarian options were also available [54]. No studies were identified that made use of any composite indicators, indexes or other tools to measure SHD outcomes

### 3.4. Identified SHD Indicators

SHD indicators were identified in two ways throughout the interventions; either by (i) addressing them through intervention activities, such as educating on food security and food waste or (ii) leveraging them as measures to evaluate the intervention impact, such as measuring plate waste post-intervention and calculating GHGE of optimized school meals [55]. This review considered SHD indicators referenced in either case (as presented in Table 2) and determined the frequency of representation of each in the reviewed articles. Similar terms that described the same SHD indicator between different studies were collated as a single indicator (for example, attitudes towards healthy food choice, environmental attitudes and nutrition knowledge were all represented as “attitudes and beliefs”).

**Figure 2:**
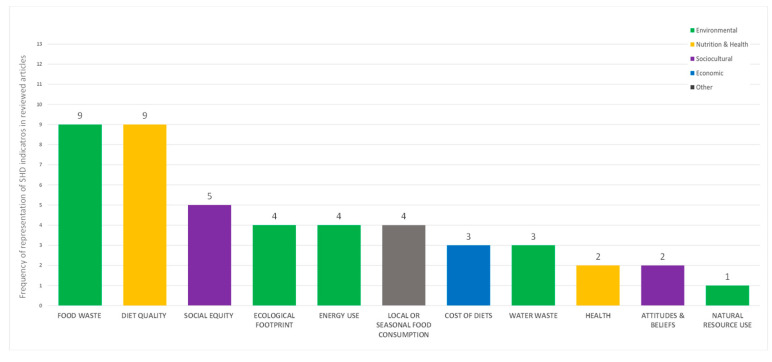
Frequency of identified SHD indicators in interventions.

Overall, we identified eleven SHD indicators, which represented all SDH dimensions, although not to the same extent (Figure 2). The environmental dimension was the most represented through five SHD indicators: (i) food waste, (ii) energy use (energy use, fossil fuel, transportation), (iii) ecological footprint (climate footprint—GHGE, food recycling, greenhouse effect, water footprint), (iv) water waste and (v) natural resource use (land use). The nutrition and health dimension followed next, represented through (i) dietary quality and (ii) health (blood results and health status), while the sociocultural and economic dimensions were underrepresented, including few citations of indicators such as social equity (cooking workload, ethical food consumption, food security and food availability) and cost of diets (affordability, meal/menu costs). Food waste and dietary quality (as adherence to the MD, daily food consumption, diet, dietary intake, nutrient dense, nutritional adequacy, vegetable consumption and vegetable intake) were the most cited indicators across all interventions, with food waste being predominantly addressed in a consistent way (cited in papers as food waste or plate waste). Other identified indicators included local or seasonal food consumption (local food procurement/consumption, local production, seasonal food consumption) and attitudes and beliefs (attitudes towards healthy food choice, environmental attitudes and nutrition knowledge).

### 3.5. Differences in Descriptions and Measuring Methods of SHD Indicators

Even when the same SHD indicator was used as a success indicator between different studies, the applied definitions and/or estimation methods varied. More specifically, we noticed several discrepancies, both in terms of defining as well as measuring food waste. The Food Resource Equity and Sustainability for Health (FRESH) study pilot used the weighted plate waste by calculating the difference between amount taken and amount eaten per pupil [60]. Boulet et. al, did not include skins, peels, cores, bones and crusts as avoidable food waste but did consider uneaten or partially eaten fruit and vegetables as avoidable food waste [65]. The OPTIMAT study that implemented an optimized meal menu to Swedish schools, detailed food waste further into five more granular terms that included kitchen waste, prepared food, serving waste, leftover food and plate waste [55].

Almost all studies assessed food or plate waste with different methods, namely visual audits [65], digital photography [61], direct weighing [56,57,58,59,60], indirect estimate of grams per pupil [54], household waste reported by parents [53] and/or in different measuring units, grams per pupil [55] and percentage based on audits or photography. This heterogeneity in methods created limitations on the direct comparison of results. Similar issues were identified for dietary quality, where some studies report outcomes for the total intake of fruit and vegetables based on adherence to the Mediterranean diet or food frequency questionnaires [53], 24 h recalls [63,64] or through weighted food waste [60], while others focused on specific food item(s) that were addressed through intervention activities, as in the case of Taniguchi and colleagues who measured dietary intake of six target vegetables in the FRESH farm-to-school curriculum [60].

## 4. Discussion

Current SHD interventions targeting primary school-aged children vary in the type of activities, the sustainability content covered and the assessment method applied to evaluate their impact. SHD indicators are included in studies either as content of educational material or as success indicators. In line with previous literature reports [34,66], the environmental and health/nutrition dimensions of SHD are the most represented, predominantly through addressing food waste and dietary quality [32,67]. Although the importance of both dimensions is clear and has been previously highlighted [68,69,70], further indicators within them or beyond are necessary to address SHD through interventions in a comprehensive way. In contrast, the social and economic dimensions are under-represented in the studies included in this review. This gap has been also reported by Nicholls and Drewnowski [32], highlighting the need to focus further on sociocultural SHD indicators, such as affordability, living environment, cultural heritage that also includes religion, and ethical aspects of food choice [71,72].

Given the composite nature of SHD, it would be expected that interventions that included clear multiple indicators would be more impactful [47]. However, based on outcomes from the most comprehensive interventions in this review, their overall effect remains unclear. For example, the FRESH study, which engaged both parents and children in school garden and curriculum-based activities that incorporated multiple SHD indicators, found no significant differences in perceived food security, food waste and dietary intake [60]. The work of Boulet et al. similarly involved both parents and children in their activities and found a 35% reduction of avoidable food waste on pooled results and higher rates of at-home children involvement in choosing and making food for school after the intervention [65]. In their modeling meal optimization study, OPTIMAT researchers included GHGE, nutrient adequacy and cost in their model, which resulted in a food list that was 40% lower in GHGE, met all nutrient recommendations for school meals and costed 11% less compared to baseline; however, food waste and consumption meal satisfaction did not significantly change [55]. Outcomes of the Healthy Children, Healthy Planet study showed that adherence to the Mediterranean Diet was not significantly affected by the intervention, although there was a significant increase in fruit consumption. In addition, the intervention group threw away less fresh fruit and bread after the 11-week intervention period [53].

Comparing results even between the most comprehensive studies is challenging, for two main reasons. First, collected data and approaches were different, as some studies did not consider direct input from children, such as the HCHP study where parents answered questionnaires on behalf of their children [53] and the OPTIMAT study which demonstrated the feasibility of their optimized meal menus mainly using linear modelling [55]. Secondly, in cases where the same indicator was addressed, studies used different definitions, measuring units and/or calculation methods making head-to-head outcome comparisons a challenging task; both food waste and dietary quality made for clear cases on these issues throughout this review, despite common reference systems, such as the European Commission’s 2019 decision for the uniform measurement of food waste levels in different environments [73]. Additionally, dietary quality within SHD is usually associated with the environmental impact of a plant-based model intake [74,75,76,77], but more composite indicators such as nutritional footprint [78], or affordable nutrient density have been proposed [35]. At present, there appears to be no dietary metric capturing all aspects of SHD [34] and the relationship between diet quality and environmental sustainability depends on how diet quality is measured [79]. This reinforces the need to harmonize indicators and their descriptions, as well as standardize measuring and/or assessment tools for SHD [28,29,30,31,33,34].

Finally, although some studies incorporated more than one indicator to evaluate their intervention effectiveness, no interventions leveraged any composite tool or index to assess adherence to SHD. Proposed methodologies that provide comprehensive approaches in measuring SHD may be key for addressing future interventions. Examples such as the SHED [36], the PHDI [37] or the WISH score [38] could provide a tangible solution for intervention studies [33].

Although varying in design and focus, SHD interventions including children have been implemented in several different countries across the world, proof of the topic’s relevance on a global level. However, it is interesting to note that within this review most of the interventions (seven out of thirteen) have been implemented in European countries. This could be attributed to the emerging EU policy strategies that highlight sustainability in recent years, and therefore reinforce the importance of harmonized policy and research efforts.

## 5. Conclusions

The majority of SHD interventions targeting school-aged children to date have been focusing predominantly on food waste and plant-based dietary quality as the primary outcomes or content of activities. Although both are critical, the environmental and nutritional dimensions of SHD are overrepresented, at the expense of the sociocultural and economic pillars. Additionally, there is often a lack of consistency in definitions and/or methods when measuring common SHD indicators, making the intervention impact unclear.

To adequately address this challenge, there is a need for extensive, composite metrics and methodological approaches to SHD. The role of researchers and policymakers is critical in defining a clearer scope and definition for SHD by determining robust indicators. In order to achieve a true population shift towards SHD, adopted practices should reflect all aspects of Sustainable Food Systems in a consistent way and include precise and clear SHD indicators in new educational and public policy material. This will enable the development of monitoring tools and updated guidelines, targeted at researchers as well as consumers, and therefore promote SHD activities. Therefore, future interventions that include primary school-aged children should (i) address a greater scope of SHD indicators and their respective dimensions (ii) apply harmonized, composite tools and standardized measuring methods after assessing specific target population needs and (iii) leverage modern digital tools to maximize impact for the community and guide future areas of focus for research and policy development.

## Figures and Tables

**Table 1:** Characteristics of included intervention studies (*n* = 13).

Authors, Year	Country	Study Design; Acronym	Sample Details (Schools or Communities; Children)	Age (Group or Mean Years Old) or School Grade	Gender Count or % (Male/Female)	Duration
Antón-Peset et al., 2021 [58]	Spain	MM with One group PPD; HPHY	1 school; 25 children	9–10	13/12	3 weeks
Boulet et al., 2022 [65]	Australia	MM with One group PPD	5 schools; 755 children	5–12	N/A	6 weeks
Colombo et al., 2020 [55]	Sweden	One group PPD; OPTIMAT ™	3 schools; 1635 children in total	10–12	N/A	4 weeks
Costarelli et al., 2021 [53]	Greece	RCT; HCHP	4 schools (2 primary and 2 kindergartens); 290 students (230 intervention, 60 control)	5–11	N/A	11 weeks
Lee et al., 2016 [62]	USA	Non-RCT	1 school; 26 children (16 intervention, 10 control)	11–13; middle school	N/A	80 min, 90 min
Lombardini et al., 2013 [54]	Finland	Non-RCT	10 schools; 38 children (26–28 intervention, 6–10 control)	7–13	N/A	11 days, 23 days
Marques et al., 2022 [59]	Portugal	One group PPD	1 school; 109 children	N/A; primary school	N/A	30 min
Martins et al., 2016 [56]	Portugal	RCT	3 schools; 147 children (25 intervention A, 55 intervention B, 28 control)	9.3; primary school	45/38	6 h, 1 h
Prescott et al., 2019 [61]	USA	MM with CT	2 schools; 918 children (697 intervention, 221 control)	11–13; middle school	361/336	6 months
Schereinemachers et al., 2017 [63]	Bhutan	RCT	18 schools (9 intervention, 9 control); 468 children (235 intervention, 233 control)	9–15	54.5%/45.5%	6–8 months
Sekiyama et al., 2017 [64]	Indonesia	One group PPD	1 school; 68 children	9.6 mean; elementary school	43/65	1 month
Taniguchi et al., 2022 [60]	USA	RCT; FRESH	2 interventions and 2 control communities; 193 children (106 intervention, 87 control)	3–6	51/55	6 months
Vidal-Mones et al., 2022 [57]	Spain	MM with One group PPD	5 schools (1 pilot, 4 case studies); 526 children	3–18	N/A	20 days

FRESH: Families Reporting Every Step to Health, HCHP: Healthy Children, Healthy Planet, HPHY: Healthy Planet, Healthy Youth, MM: Mixed Methods, Non-RCT: Non-Randomized Controlled Trial, One group PPD: One group Pretest Posttest Design, RCT: Randomized Controlled Trial.

**Table 2:** Intervention activities, impact evaluation methods and identified SHD indicators.

Authors, Year	SHD Indicator(s)	Intervention Activities	Collected Data	Impact Evaluation (Change Post-Intervention)
Antón-Peset et al., 2021 [58]	food waste, water waste, energy use, recycling, diet, social food injustice, greenhouse effect, water footprint	awareness activities classroom didactic sessions	attitudes and knowledge plate waste	questionnaire on attitudes/knowledge plate waste (direct weighing)
Boulet et al., 2022 [65]	food waste	lessons for students parent engagement hands on workshops “make your lunch” day	food behavior food waste	online survey (only ages 8–12) food waste (visual audits)
Colombo et al., 2020 [55]	daily food consumption, climate footprint—GHGE (kg CO_2_ eq/kg), nutritional adequacy, affordable, food waste	replacement of school menus with optimized sustainable meals information to parents and school	meal satisfaction plate waste food consumption	meal satisfaction plate waste per pupil (grams) food consumption per pupil (grams) SHD meal indicators (GHGE, cost, nutritional adequacy)
Costarelli et al., 2021 [53]	ecological footprint, food waste, water waste, adherence to the MD, seasonal food consumption, food recycling, ethical food consumption, household waste	teacher training sessions parents’ educational session teacher feedback meeting educational material for teachers, pupils and parents	anthropometric adherence to MD FFQ physical activity levels eating behavior food waste behavior	questionnaire food waste (household level, reported by parents)
Lee et al., 2016 [62]	attitudes towards healthy food choices, seasonal food consumption, local food procurement/consumption	classroom session field trip to local community farm	demographic behavioral intention and attitude historical social norm scores	behavioral intention & attitude historical social norm scores
Lombardini et al., 2013 [54]	food waste	forced vegetarian day	food taken (estimate; total amount by participants) plate waste (estimate; total amount by participants) vegetable share (%)	participation in FVD food taken (estimate; total amount by participants) plate waste (estimate; total amount by participants) vegetable share (%)
Marques et al., 2022 [59]	plate waste, vegetable consumption	nutritionist session interactive play	prepared vegetables vegetable waste	distributed vegetable weight consumed vegetable weight (per capita) vegetable waste (weighing) leftover index plate waste index
Martins et al., 2016 [56]	food waste, social, economic parameters of food systems, nutrition knowledge	Intervention A (children) -nutrition education, impact of food waste Intervention B (teachers)—impact of food waste, posters	sociodemographic plate waste	plate waste (via direct weighing)
Prescott et al., 2019 [61]	natural resources (land use), environmental attitudes, food waste, energy use (transportation, fossil fuel), dietary intake (vegetables)	educational sessions teacher training salad bar plate waste assessment student posters	demographic attitudes and knowledge on food systems food waste	A 46-item survey FTTB vegetable consumption plate waste (digital photography)
Schereinemachers et al., 2017 [63]	vegetable consumption, local production, nutrient-dense	education (classroom sessions) school gardening student activities on lessons learned (co-creation)	anthropometric dietary intake (24 h recalls) knowledge on sustainable agriculture knowledge on food and nutrition awareness on fruit and vegetables	anthropometric dietary intake (focused FV) knowledge on sustainable agriculture knowledge on food and nutrition awareness of fruit and vegetables
Sekiyama et al., 2017 [64]	food availability, cooking workload, meal/menu cost	sustainable school lunch meals	anthropometric socioeconomic dietary intake (24 h recalls) blood sampling food-related knowledge, behavior	blood results (hemoglobin and hematocrit) anthropometric (BMI) dietary intake
Taniguchi et al., 2022 [60]	dietary intake, plate waste, health status, food security	nutrition curriculum school garden parent curriculum F2S menu modifications	anthropometric dietary intake (through food waste) willingness to try food insecurity	dietary intake (weighted plate waste) BMI willingness to try/preference food insecurity (survey)
Vidal-Mones et al., 2022 [57]	food waste	co-creation and implementation of nudging strategies plate waste at school canteens	plate waste	plate waste (via direct weighing)

ARD: Average Relative Deviation, BMI: Body Mass Index, FFQ: Food Frequency Questionnaire, F2S: Farm to School, FRESH: Food Resource Equity and Sustainability for Health, FV: Fruit & Vegetables, FVD: Forced Vegetarian Day, FTTB: Farm to Table and Beyond, GHGE: Greenhouse Gas Emissions, MD: Mediterranean Diet.

## Data Availability

Not applicable.

## Supplementary Materials

The following supporting information can be downloaded at: https://www.mdpi.com/article/10.3390/nu15112460/s1, PRISMA 2022 Checklist.

## Appendix A. MMAT Quality Assessment Outcomes

**Authors, Year**	**Country**	**Study Design; Acronym**	**MMAT Category**	**Quality**
Antón-Peset et al., 2021 [58]	Spain	MM with One group PPD; HPHY	Mixed Methods Studies	****
Boulet et al., 2022 [65]	Australia	MM with One group PPD	Mixed Methods Studies	*****
Colombo et al., 2020 [55]	Sweden	One group PPD; OPTIMAT ™	Non Randomized Studies	****
Costarelli et al., 2021 [53]	Greece	RCT; HCHP	Randomized Controlled Trials	***
Lee et al., 2016 [62]	USA	Non-RCT	Non Randomized Studies	***
Lombardini et al., 2013 [54]	Finland	Non-RCT	Non Randomized Studies	****
Marques et al., 2022 [59]	Portugal	One group PPD	Non Randomized Studies	***
Martins et al., 2016 [56]	Portugal	RCT	Randomized Controlled Trials	****
Prescott et al., 2019 [61]	USA	MM with CT	Mixed Methods Studies	****
Schereinemachers et al., 2017 [63]	Bhutan	RCT	Randomized Controlled Trials	*****
Sekiyama et al., 2017 [64]	Indonesia	One group PPD	Non Randomized Studies	***
Taniguchi et al., 2022 [60]	USA	RCT; FRESH	Randomized Controlled Trials	****
Vidal-Mones et al., 2022 [57]	Spain	MM with One group PPD	Mixed Methods Studies	***
